# Does High-Dose Thromboprophylaxis Improve Outcomes in COVID-19 Patients? A Meta-analysis of Comparative Studies

**DOI:** 10.1055/a-1930-6492

**Published:** 2022-10-19

**Authors:** Maha A. T. Elsebaie, Binav Baral, Mai Elsebaie, Trilok Shrivastava, Catherine Weir, Dennis Kumi, Noah W. Birch

**Affiliations:** 1 Department of Hematology and Medical Oncology, John H. Stroger, Jr. Hospital of Cook County, Chicago, Illinois, United States; 2 Faculty of Medicine, Ain Shams University, Cairo, Egypt

**Keywords:** thrombosis, bleeding, COVID-19, thromboprophylaxis, therapeutic anticoagulation

## Abstract

**Background** Thromboembolism remains a detrimental complication of novel coronavirus disease (COVID-19) despite the use of prophylactic doses of anticoagulation

**Objectives** This study aimed to compare different thromboprophylaxis strategies in COVID-19 patients

**Methods** We conducted a systematic database search until June 30, 2022. Eligible studies were randomized (RCTs) and nonrandomized studies that compared prophylactic to intermediate or therapeutic doses of anticoagulation in adult patients with COVID-19, admitted to general wards or intensive care unit (ICU). Primary outcomes were mortality, thromboembolism, and bleeding events. Data are analyzed separately in RCTs and non-RCTs and in ICU and non-ICU patients.

**Results.** We identified 682 studies and included 53 eligible studies. Therapeutic anticoagulation showed no mortality benefit over prophylactic anticoagulation in four RCTs (odds ratio [OR] = 0.67, 95% confidence interval [CI], 0.18–2.54). Therapeutic anticoagulation didn't improve mortality in ICU or non-ICU patients. Risk of thromboembolism was significantly lower among non-ICU patients who received enhanced (therapeutic/intermediate) anticoagulation (OR = 0.21, 95% CI, 0.06–0.74). Two additional RCTs (Multiplatform Trial and HEP-COVID), not included in quantitative meta-analysis, analyzed non-ICU patients, and reported a similar benefit with therapeutic-dose anticoagulation. Therapeutic anticoagulation was associated with a significantly higher risk of bleeding events among non-randomized studies (OR = 3.45, 95% CI, 2.32–5.13). Among RCTs, although patients who received therapeutic-dose anticoagulation had higher numbers of bleeding events, these differences were not statistically significant. Studies comparing prophylactic and intermediate-dose anticoagulation showed no differences in primary outcomes.

**Conclusion** There is a lack of mortality benefit with therapeutic-dose over prophylactic-dose anticoagulation in ICU and non-ICU COVID-19 patients. Therapeutic anticoagulation significantly decreased risk of thromboembolism risk in some of the available RCTs, especially among non-ICU patients. This potential benefit, however, may be counter balanced by higher risk of bleeding. Individualized assessment of patient's bleeding risk will ultimately impact the true clinical benefit of anticoagulation in each patient. Finally, we found no mortality or morbidity benefit with intermediate-dose anticoagulation.

## Introduction

Novel coronavirus disease 2019 or severe acute respiratory syndrome-coronavirus-2 (COVID-19 or SARS-CoV-2) is a global pandemic leading to widespread infection and mortality. The precise mechanism of thromboembolism in COVID-19 remains unclear, although experts in the field have proposed various explanations. Endothelial damage, one of the critical components of the Virchow triad, seems to be the primary driver of thrombosis. 1 The direct viral endothelial infection causes endothelial activation, aided by the “cytokine storm” caused by COVID-19 (mainly due to interleukin [IL]-1, IL-6, and tumor necrosis factor [TNF]-α). This results in activation of fibrinogen and recruitment of leukocytes into the subendothelial layer increasing inflammation. 2

The most common causes of death in COVID-19 patients are thromboembolism, cytokine storm, and acute respiratory distress syndrome. 3 Anticoagulation therapy was shown to significantly reduce fibrin deposition, microthrombi formation, and overall mortality in COVID-19 patients. 4 Nonetheless, the optimal thromboprophylaxis regimen for COVID-19 patients is still not clear. More recently, studies have shown high rates of thromboembolism and in-hospital mortality despite standard prophylactic doses of anticoagulation.

Alerted by these findings, institutions began efforts to tailor increases in anticoagulation regimens. Explanations provided for escalating anticoagulation in COVID-19 included the relative hyperfibrinogenemia seen in COVID-19 which could mediate heparin resistance 5 and even the possible protective effect of heparin in preventing viral attachment and entry to mucosal epithelia. 6

Clinical outcomes of high-dose thromboprophylaxis have so far been conflicting, with many studies showing high bleeding risk with preemptive therapeutic doses of anticoagulation. 7 8 9 The conflicting observations and sporadic data are seemingly contradictory and can reasonably be expected in a novel global pandemic of these proportions. Therefore, we conducted an in-depth meta-analysis aiming to assess common observations across various studies which have been conducted since the beginning of the pandemic and derive objective conclusions from the reported observations regarding the delicate balance of thrombosis and anticoagulation in COVID-19.

## Materials and Methods

### Database Search

A comprehensive search on Medline, the Cochrane COVID-19 Study Register, and Clinicaltrials.gov was performed from inception until August 31, 2021. Search terms were “anticoagulant,” “anticoagulation,” “heparin,” or “thromboprophylaxis,” AND “COVID-19” or “SARS-CoV-2.” Filters were applied to display comparative studies, clinical trials, retrospective cohort, and prospective observational studies. No language restrictions were applied. Reference lists of all included original articles, and four recent systematic reviews were hand searched. 10 11 12 13 To accommodate the rapidly evolving literature on thromboembolism in the ongoing COVID-19 pandemic, we performed an updated search (using same search terms) on Medline and Clinicaltrials.gov for randomized clinical trials published between September 1, 2021, and June 30, 2022.

### Study Selection

Eligible studies were randomized and nonrandomized studies with the following features: (1) comparing prophylactic to intermediate or therapeutic doses of anticoagulation, (2) anticoagulation used for thromboprophylaxis, and (3) population were adults (>18 years of age) with a confirmed diagnosis of COVID-19, admitted to the general wards or intensive care unit (ICU). Primary outcomes were mortality rates, risk of thromboembolism (e.g., deep venous thrombosis [DVT], pulmonary embolism [PE], stroke, or myocardial infarction [MI]), and rates of bleeding events. Secondary outcomes were length of hospital stay (LOS) and organ support-free days (OS-free). These secondary outcomes were chosen as surrogates for morbidity. Organ support was defined as invasive or noninvasive mechanical ventilation, high-flow nasal oxygen, vasopressor therapy, or extracorporeal membrane oxygenation support.

The following studies were excluded: (1) noncomparative studies, (2) no prophylactic dose arm, (3) not studying primary outcomes of interest, (4) outcomes were not reported separately for each treatment group, (5) studies primarily focused on the role of thrombolysis or monoclonal antibodies, (6) studies in pediatric patients, and (7) case reports and small case series (e.g., studies with less than 20 patients).

### Data Extraction

Three authors (M.A.E., B.B., and T.S.) screened titles and abstracts for eligibility. Potentially eligible articles were then checked for thromboprophylaxis arms and outcomes of interest. Finally, the authors cross-checked the data and any discrepancies were resolved by discussion. Database search and data extraction were conducted and presented according to the Preferred Reporting Items for Systematic Reviews and Meta-analysis (PRISMA) guidelines. 14

Data were extracted into a standardized spreadsheet and included the following: study design, year of publication, country of origin, patient inclusion criteria, median age of patients, median body mass index (BMI), percentage of male patients, percentage of ICU admissions, patient comorbidities, length of hospital stay, anticoagulation strategies, indications for higher than prophylactic doses of anticoagulation, concomitant therapy (e.g., antiplatelet and antiviral therapy), and outcome data.

Thromboprophylaxis strategies were divided into the following three categories: (1) prophylactic dose (enoxaparin 30–40 mg/day or equivalent doses of another LMWH, fondaparinux, unfractionated heparin, or direct oral anticoagulant [DOAC]), (2) intermediate dose (doses higher than prophylactic but less than therapeutic dose, usually weight-adjusted or double prophylactic dose), and (3) therapeutic dose: enoxaparin 1 mg/kg twice daily or 1.5 mg/kg once daily or equivalent doses of other anticoagulants.

### Quality Assessment

For nonrandomized cohort studies, we used the Newcastle–Ottawa scale (NOS) to assess the quality of studies. 15 In this scale, a maximum of 8 points can be assigned for the highest quality study in three domains: selection of study groups, comparability between groups, and adequacy of outcome assessment and follow-up. Studies judged as medium (3–5 points) or high (>5 points) quality were included in quantitative analysis.

For randomized controlled trials (RCTs), we used the Cochrane Collaboration's tool to assess bias risk. This scale covers seven bias domains: selection bias (two domains), performance bias, detection bias, attrition bias, reporting bias, and other potential sources of bias. Two independent authors performed quality assessments (B.B. and M.E.), and discrepancies were resolved by discussion.

### Statistical Analysis

All meta-analyses were performed in a random-effects model using the Mantel–Haenszel method. 16 For dichotomous outcomes (e.g., mortality), we calculated pooled odds ratios (ORs) and 95% confidence intervals (CIs). For continuous outcomes (e.g., LOS), we calculated standardized mean difference (Std. mean diff.) and standard error (SE). We contacted corresponding authors of studies that reported LOS or OS-free days in medians instead of means. For the two studies that we could not obtain summary data in means, we used the method published by Wan et al to estimate the sample mean and standard deviation using the study sample size, median, and interquartile range. 17 18 19

All results are shown separately for nonrandomized (text in black within figures) and randomized studies (text in red within figures). We also performed subgroup analyses for primary outcomes according to ICU admission status (ICU vs. Non-ICU patients).

We conducted a meta-regression for covariates affecting thromboembolism and bleeding risk, among the nonrandomized studies that compared prophylactic and therapeutic anticoagulation. The small number of randomized studies did not allow for regression analysis. Coefficient ( *β* ) indicates both the direction and magnitude of association between the covariate and effect size. For instance, a negative *β* is associated with lower OR. The *R* ^2^ analog represents the total in-between study variance explained by the model. Covariates were chosen for the metaregression model if they were clinically meaningful or significantly associated with the effect size.

Heterogeneity was assessed using the Cochran *Χ* ^2^ test (Q) and the *I* ^2^ statistics. 20 A Cochrane *Χ* ^2^ test *p* -value of <0.05 was considered statistically significant for interstudy heterogeneity. An *I* ^2^ value of 25% represents insignificant heterogeneity, 26 to 50% low heterogeneity, 51 to 75% moderate heterogeneity, and >75% high heterogeneity. Publication bias was assessed by visual inspection of funnel plots. All analyses were performed using Comprehensive Meta-analysis V3 and RevMan V5 software.

## Results

### Study Selection and Characteristics

**Fig. 1:**
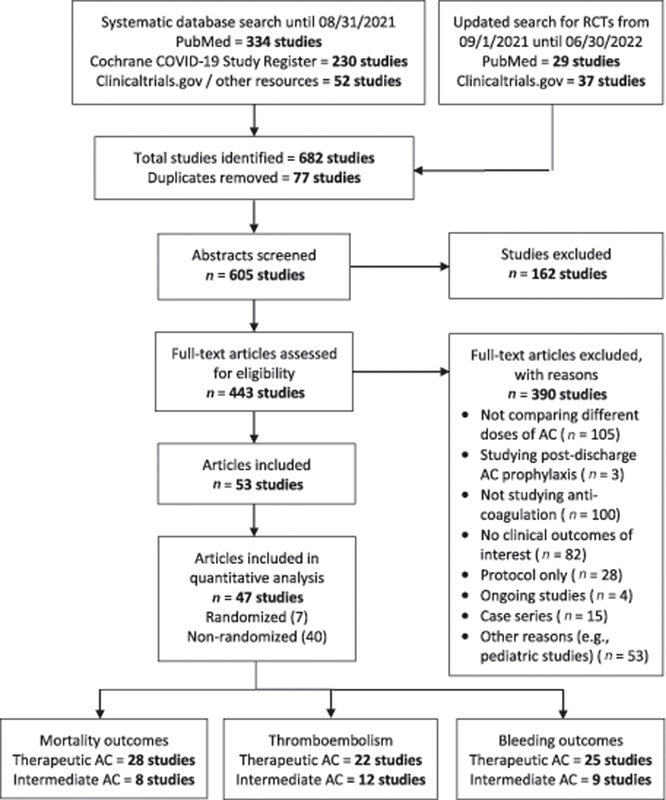
Prisma flow chart.

Systematic database search until June 30, 2022, identified 682 studies from Medline, the Cochrane COVID-19 Study Register, and other sources. 443 full-text articles were screened for eligibility, and 53 were found eligible, Fig. 1 . Reasons for exclusion are summarized in Fig. 1 . Seven studies were excluded because the proportion of patients in the intermediate/therapeutic dose arm was deficient, precluding meaningful comparisons.

Characteristics of included studies are summarized in Supplementary Table S1 . There were 40 nonrandomized retrospective and prospective cohort studies and 10 RCTs. 4 5 7 8 9 17 18 19 21 22 23 24 25 26 27 28 29 30 31 32 33 34 35 36 37 38 39 40 41 42 43 44 45 46 47 48 49 50 51 52 53 54 55 56 57 58 59 60 61 62 63 In three of the included RCTs, data from patients who received prophylactic and intermediate doses were pooled into one arm against therapeutic-dose anticoagulation. 18 60 We contacted the corresponding authors to obtain subgroup data in prophylactic and intermediate-dose arms separately; however, they were not able to accommodate our requests. Hence, results from the two multiplatform trials in critically ill and noncritically ill patients, and the HEP-COVID trial are excluded from the quantitative meta-analyses and discussed separately. Results from these trials are summarized in Table 1 .

**Table 1:** Summary of results from the multiplatform and HEP-COVID trials

Randomized trial	Mortality outcomes			Thromboembolism			Bleeding outcomes			
	Therapeutic (E/total)	Nontherapeutic (E/total)	OR (95% CI)	Therapeutic (E/total)	Nontherapeutic (E/total)	OR (95% CI)	Therapeutic (E/total)	Nontherapeutic (E/total)	OR (95% CI)
Multiplatform trial REMAP-CAP a									
ICU patients (100%)	199/534	200/564	1.08 (0.85–1.38)	38/530	62/559	0.62 (0.41–0.94)	20/529	13/562	1.66 (0.82–3.37)
Multiplatform trial ATTACC b									
Non-ICU patients (100%)	86/1,180	86/1,046	0.88 (0.64–1.20)	16/1,180	28/1,046	0.50 (0.27–0.93)	22/1,180	9/1,047	2.19 (1.00–4.78)
HEP-COVID trial c									
Total	25/129	31/124	0.72 (0.40–1.31)	14/129	36/124	0.30 (0.15–0.59)	6/129	2/124	2.98 (0.59–15.03)
ICU patients (32.8%)	16/45	15/38	0.85 (0.35–2.06)	7/45	11/38	0.45 (0.16–1.32)	4/45	0/38	8.35 (0.44–160.24)
Non-ICU patients (67.2%)	9/84	16/86	0.53 (0.22–1.26)	7/84	26/86	0.21 (0.09–0.52)	2/84	2/86	1.02 (0.14–7.44)

Abbreviations: E/total: events/total number of patients. OR: odds ratio. CI: confidence Interval. ICU: intensive care unit.

a Nontherapeutic dose arm included 41% patients with prophylactic dose and 51% patients with intermediate dose.

b Nontherapeutic dose arm included 71.7% patients with prophylactic dose and 26.5% patients with intermediate dose.

c Nontherapeutic dose arm included 61.3% patients with prophylactic dose and 38.7% patients with intermediate dose.

The studies by Pierce-William et al, Al-samkari et al 2020, and Koleilat et al were of insufficient quality to be included in the quantitative analysis. 3 64 65 At the time of this publication, the study by Trinh et al was still a non-peer-reviewed pre-print.

### Indications for Therapeutic Anticoagulation

The rationale for using therapeutic anticoagulation varied between nonrandomized studies. In six studies, patients were on chronic therapeutic anticoagulation for non-COVID-19-related reasons (e.g., atrial fibrillation). Thus, therapeutic anticoagulation was continued after hospitalization. 22 29 33 35 41 46 In 13 studies, the decision was left to the treating physicians and was based on clinical (e.g., age, body mass index, and comorbidities), laboratory (e.g., D-dimer level and C-reactive protein), and radiological findings that indicated a higher risk of mortality. 7 28 30 32 36 40 42 56 In six studies, anticoagulation doses were based on locally or nationally adapted thromboprophylaxis guidelines 4 8 23 34 37 44 that continued to evolve as more literature became available on COVID-19. Many of the remaining articles mentioned no clear criteria for choosing therapeutic anticoagulation.

### Therapeutic Dose Anticoagulation

#### Mortality Outcomes

**Fig. 2:**
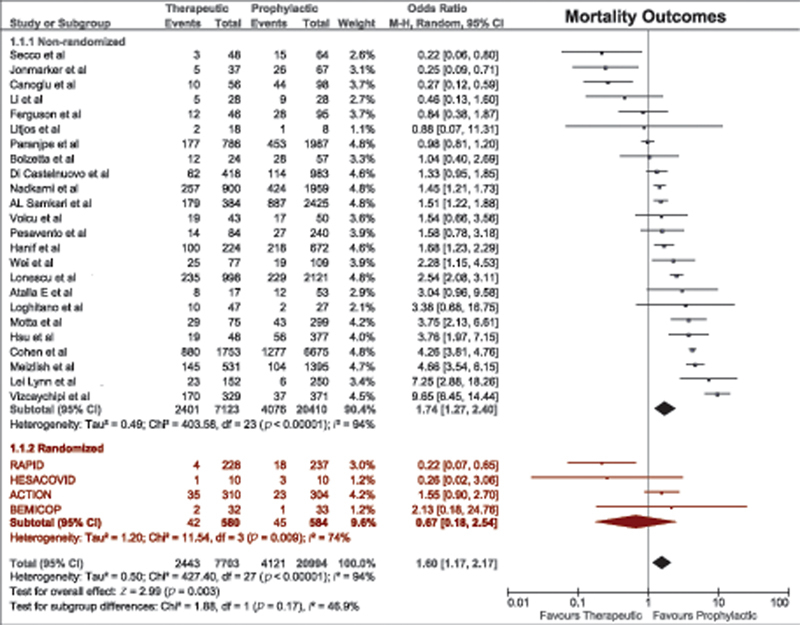
Forest plot for mortality outcomes with therapeutic versus prophylactic doses of anticoagulation. CI, confidence interval.

A total of 28 studies compared mortality between therapeutic-dose anticoagulation and prophylactic-dose anticoagulation. Patients receiving prophylactic anticoagulation had better survival than those treated with therapeutic anticoagulation (OR = 1.60, 95% CI, 1.17–2.17; Fig. 2 ). However, this effect was lost when the analysis was limited only to RCTs (OR = 0.67, 95% CI, 0.18–2.54), the *p* -value for test for subgroup differences of 0.17.

Similarly, in the two multiplatform trials in critically ill and noncritically ill patients, and the HEP-COVID trial, there were no significant differences in mortality between patients who received therapeutic and nontherapeutic anticoagulation, Table 1 .

#### Thromboembolism

**Fig. 3:**
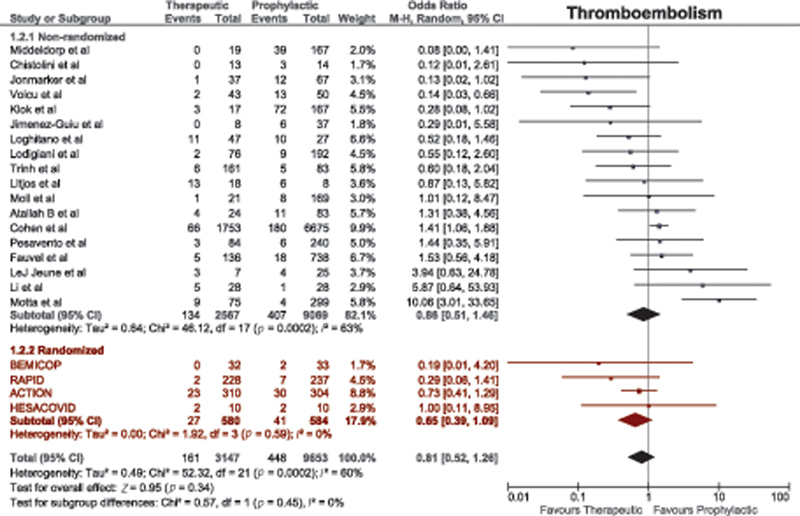
Forest plot for thromboembolism with therapeutic versus prophylactic doses of anticoagulation. CI, confidence interval.

A total of 22 studies compared thromboembolism between therapeutic-dose anticoagulation and prophylactic-dose anticoagulation. There was no difference in risk of thromboembolism among nonrandomized studies. There was a nonsignificant trend toward lower risk of thromboembolism with therapeutic-dose anticoagulation among RCTs (OR = 0.65, 95% CI, 0.39–1.09; Fig. 3 ).

In the two multiplatform trials in critically ill and noncritically ill patients, and the HEP-COVID trial, therapeutic-dose anticoagulation significantly decreased risk of thromboembolism compared with non-therapeutic anticoagulation: OR = 0.62, 95% CI, 0.41–0.94; OR = 0.50, 95% CI, 0.27–0.93; and OR = 0.30, 95% CI, 0.15–0.59, respectively ( Table 1 ).

#### Bleeding Outcomes

**Fig. 4:**
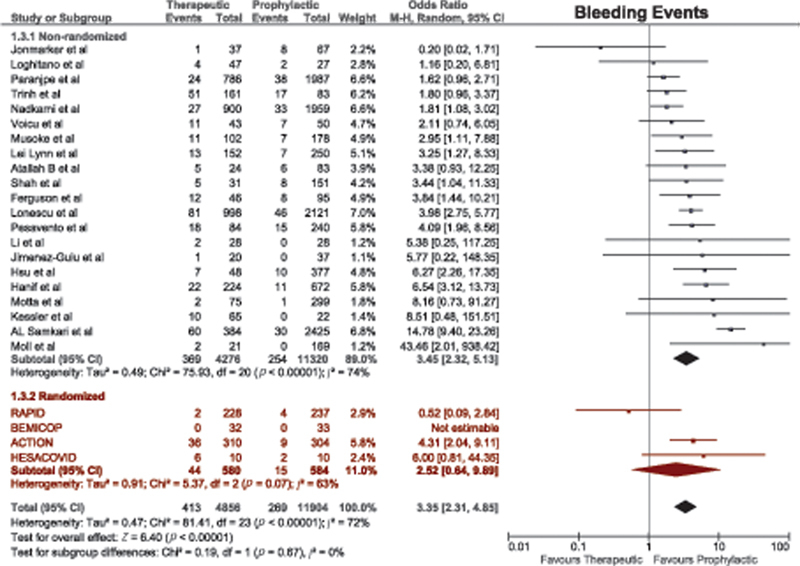
Forest plot for bleeding events with therapeutic versus prophylactic doses of anticoagulation. CI, confidence interval.

A total of 25 studies compared bleeding outcomes between therapeutic-dose and prophylactic-dose anticoagulation. Therapeutic anticoagulation was associated with a significantly higher risk of bleeding events among nonrandomized studies (OR = 3.45, 95% CI, 2.32–5.13). However, this effect was lost among RCTs ( Fig. 4 ). Although patients who received therapeutic-dose anticoagulation in the two multiplatform trials and the HEP-COVID trial had higher numbers of bleeding events, these differences were not statistically significant ( Table 1 ).

### Intermediate Dose Anticoagulation

#### Mortality Outcomes

Eight studies compared intermediate and prophylactic-dose anticoagulation and showed no significant difference in mortality outcomes between the two strategies (OR = 1.07, 95% CI, 0.55–2.08; Supplementary Fig. S1 ).

#### Thromboembolism

Twelve studies compared intermediate and prophylactic-dose anticoagulation and showed that Intermediate-dose anticoagulation did not influence thromboembolism risk (OR = 1.13, 95% CI, 0.87–1.48; Supplementary Fig. S2 ).

#### Bleeding Outcomes

Bleeding risk was also similar between intermediate-dose and prophylactic-dose anticoagulation in nine studies: OR = 1.01, 95% CI, 0.72–1.41; Supplementary Fig. S3 ).

### Subgroup Analysis (Intensive Care Unit vs. Non–Intensive Care Unit Patients)

#### Intensive Care Unit Patients

**Fig. 5:**
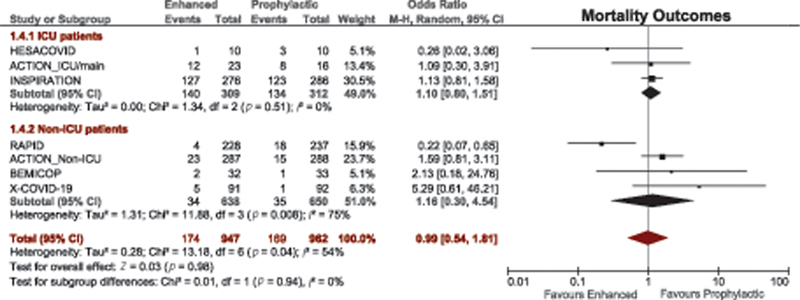
Subgroup analysis of mortality outcomes in ICU and non-ICU patients who received enhanced (therapeutic/intermediate) versus prophylactic anticoagulation. CI, confidence interval; ICU, intensive care unit.

*Mortality outcomes* : primary outcomes were specified according to ICU admission status in nine RCTs. There was no significant association between in-hospital mortality and enhanced thromboprophylaxis (therapeutic or intermediate dose) in ICU patients (OR = 1.10, 95% CI, 0.80–1.51; Fig. 5 ).

Similarly, in the multiplatform trial in critically ill patients and the subset of ICU patients in HEP-COVID trial, there were no significant differences in mortality between patients who received therapeutic and non-therapeutic anticoagulation ( Table 1 ).

**Fig. 6:**
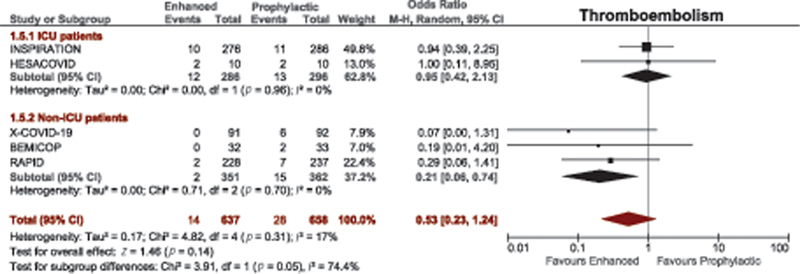
Subgroup analysis of thromboembolism in ICU and non-ICU patients who received enhanced (therapeutic/intermediate) versus prophylactic anticoagulation. CI, confidence interval; ICU, intensive care unit.

*Thromboembolism* : enhanced thromboprophylaxis compared with standard prophylactic-dose did not influence the risk of thromboembolism in ICU patients ( Fig. 6 ). Similar results were noted among the subset of ICU patients in the HEP-COVID trial (OR = 0.45, 95% CI, 0.16–1.32; Table 1 ).

**Fig. 7:**
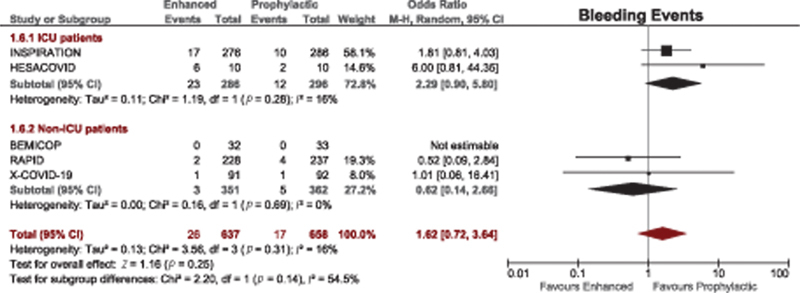
Subgroup analysis of bleeding events in ICU and non-ICU patients who received enhanced (therapeutic/intermediate) versus prophylactic anticoagulation. CI, confidence interval; ICU, intensive care unit.

*Bleeding outcomes* : there was no significant difference in risk of bleeding among ICU patients ( Fig. 7 ).

#### Non–Intensive Care Unit Patients

*Mortality outcomes* : there was no significant association between in-hospital mortality and enhanced thromboprophylaxis (therapeutic or intermediate dose) in non-ICU patients (OR = 1.16, 95% CI, 0.30–4.54; Fig. 5 ).

*Thromboembolism* : enhanced thromboprophylaxis significantly decreased the risk of thromboembolism in non-ICU patients (OR = 0.21, 95% CI, 0.06–0.74; Fig. 6 ). These findings were similar to results from the Multiplatform trial in noncritically ill patients and the subset of non-ICU patients from the HEP-COVID trial ( Table 1 ).

*Bleeding outcomes* : there was no significant difference in risk of bleeding among non-ICU patients ( Fig. 7 ).

### Metaregression Analysis

#### Thromboembolism

Supplementary Table S2 shows the univariable analysis for different study characteristics associated with thromboembolism. In a meta-regression analysis limited to percent ICU patients, percent male patients, and median age, a higher percentage of ICU patients was associated with a greater benefit from therapeutic compared with prophylactic anticoagulation ( *β* = − 0.0206, *p* = 0.029, *R* ^2^ = 0.46). However, this association between percentage of ICU patients and therapeutic anticoagulation was lost in another analysis model that included percent ICU patients, percent male patients, and median follow-up duration ( *β* = − 0.0069, *p* = 0.562, *R* ^2^ = 0.52).

#### Bleeding Events

Supplementary Table S3 shows the univariable analysis for different study characteristics associated with the risk of bleeding. In a meta-regression analysis of median BMI, median platelet count, and median fibrinogen level, a higher median platelet count was associated with a lower risk of bleeding ( *β* = − 0.086, *p* = 0.012, *R* ^2^ = 0.78) with therapeutic anticoagulation. A separate meta-regression analysis including percent of patients with respiratory disease, hypertension, or diabetes revealed a significant association between percent of patients with respiratory disease and bleeding risk ( *β* = 0.081, *p* = 0.023, *R* ^2^ = 0.67).

#### Surrogate Morbidity Outcomes

The mean LOS was significantly lower among patients who received prophylactic compared with enhanced (intermediate/therapeutic) anticoagulation in six non-randomized studies (std. mean diff. = 0.705, 95% CI, 0.27–1.13, *p* = 0.002). However, LOS was similar between the treatment groups in four RCTs (std. mean diff. = 0.11, 95% CI, −0.02 to 0.23; Supplementary Fig. S4 ). OS-free days were also similar between prophylactic and enhanced anticoagulation groups in four RCTs ( Supplementary Table S4 Supplementary Fig. S5 ).

#### Risk of Bias Assessment

The risk of bias among non-randomized studies is summarized in Supplementary Table S4 . Supplementary Fig. S6 demonstrates the risk of bias summary among ten included RCTs using the Cochrane Collaboration tool. Based on visual inspection of funnel plots, there was no evidence of publication bias among primary or secondary outcome comparisons.

## Discussion

COVID-19 infection has become a global pandemic of immense proportions. It has generated much uncertainty and strain on health care systems while impacting a variety of organ systems in patients worldwide. While treatment options have been developed including multiple prophylactic vaccines, the question of adequate thromboprophylaxis remains unanswered after more than two years of intensive trial and error.

Thromboembolism is a detrimental complication of COVID-19, with overall rates as high as 21% in hospitalized patients and 31% in critically ill patients. 35 41 In this meta-analysis comparing different thromboprophylaxis strategies, we highlight the following observations: (1) lack of survival benefit with therapeutic compared with prophylactic anticoagulation in both ICU and non-ICU patients; (2) Therapeutic anticoagulation significantly decreased risk of thromboembolism in some of the available RCTs, especially among non-ICU patients; (3) bleeding events occurred more frequently with therapeutic anticoagulation in nonrandomized and randomized studies, although numbers did not reach statistical significance among RCTs; and (4) lack of mortality or morbidity benefit with intermediate-dose anticoagulation.

There appears to be a discrepancy between nonrandomized and randomized data regarding the mortality benefit of therapeutic anticoagulation. In the nonrandomized studies, although there was a statistically significant difference in mortality between prophylactic and therapeutic anticoagulation, this difference likely reflects the severity of illness rather than the true therapeutic effect of anticoagulation: therapeutic anticoagulation was likely reserved for managing more severe disease, and hence reflected the worse prognosis associated with severe infections. 51

This difference in mortality between therapeutic and prophylactic anticoagulation disappeared once adjusted for disease severity by analyzing ICU or non-ICU patients separately. A recent RCT of more than 1,000 ICU patients also highlighted the lack of survival benefit with therapeutic anticoagulation. 18 In the few exceptions that reported a survival benefit with therapeutic anticoagulation in ICU patients, results were limited by their observational nature, the relatively short duration of therapeutic anticoagulation, and frequent adjustments in anticoagulation dosing. 4 36 51 In one study, the survival benefit from therapeutic anticoagulation disappeared after 4 days of anticoagulation. Hence the survival benefit highlighted in some observational studies may represent a timing bias.

The mortality benefit from therapeutic anticoagulation in non-ICU patients was recently explored in an RCT of >2,000 noncritically ill patients. 18 In this study, therapeutic heparin increased the probability of survival. It is possible to conclude that the antithrombotic and anti-inflammatory effects of therapeutic anticoagulation may not be enough to alter the course of patients with severe disease (i.e., those hospitalized in the ICU) but could offer therapeutic benefit to those with mild or moderate disease.

While not seen in mortality outcomes, there does appear to be a morbidity benefit in preventing thromboembolism with therapeutic anticoagulation. This benefit of decreasing thromboembolism risk is especially seen in non-ICU patients. As described above, however, mortality benefit is not significantly improved with therapeutic anticoagulation. 18 This discordance may be explained by the noticeable trend toward higher risk of bleeding with therapeutic-dose anticoagulation among published data. Taken together, this meta-analysis demonstrates that the potential benefits of preventing thromboembolism with therapeutic anticoagulation in COVID19-positive patients may be counter-balanced by factors that impact a patient's individual risk of bleeding.

We performed a meta-regression that revealed that certain patient characteristics may be associated with a higher risk of bleeding. These characteristics include low platelet count and patient comorbidities including underlying respiratory disease. Patients with chronic lung disease are at higher risk for severe pulmonary inflammation, and therefore have a higher risk of alveolar hemorrhage with therapeutic anticoagulation. 18 Together, this meta-analysis reminds treating providers to perform a nuanced assessment of each patient when determining whether to consider therapeutic anticoagulation in COVID-19-positive patients. It will be important to consider not just the severity of each person's illness but also to assess each patient's individual bleeding risk in the context of their laboratory parameters and comorbid medical conditions.

Around 15 retrospective studies and three RCTs have explored the benefit of intermediate-dose anticoagulation in COVID-19 patients. Our analysis, however, has shown no tangible benefit in either morbidity or mortality for the use of intermediate dosing. Jonmarker et al first reported the use of intermediate-dose anticoagulation and were unable to show a benefit over prophylactic anticoagulation. This was followed by multiple studies with mixed results. 22 32 34 These efforts were limited by the lack of a standardized definition for what constitutes “intermediate anticoagulation.” Furthermore, the sample size was suboptimal, and there was a high frequency of cross-over between anticoagulation regimens in these studies. We conclude that the combination of inconsistent methodology, low statistical power, and lack of precision in outcomes of intermediate-dose anticoagulation undermines its clinical utility currently and further muddies the already cloudy waters of anticoagulation in COVID-19.

## Limitations

To the best of our knowledge, this is the most in-depth meta-analysis of the comparative randomized and nonrandomized studies which explore thromboprophylaxis in COVID-19 patients. Nonetheless, our study has several limitations, including the small number of RCTs and the heterogeneity among observational studies. We analyzed ICU and non-ICU patients separately and conducted a meta-regression analysis of different study characteristics to address this heterogeneity between studies. It is also worth mentioning that many of the included studies were published before distributing the first COVID-19 vaccine in December 2020. Hence, some of included studies reflect outcomes from the pre-vaccination era. Nevertheless, more than 30% of the U.S. and worldwide populations are unvaccinated and the hospitalization rate of the unvaccinated are outpacing those of vaccinated patients. Given the persistent worldwide burden of COVID-19 and continued hospitalizations for acute infections, this analysis remains highly relevant.

## Conclusion and Future Directions

The data collected since 2019, while limited by a small number of RCTs, have revealed important points in the pursuit of adequate thromboprophylaxis in COVID-19 patients. Available literature argues against the mortality benefit from therapeutic anticoagulation. The benefit of therapeutic anticoagulation in preventing thromboembolism must be weighed against each patient's risk of bleeding. Future studies are encouraged to (1) explore predictors of major bleeding and subsequent risk of death, and (2) identify a subset of COVID-19 patients who could safely benefit from therapeutic anticoagulation with the least risk of bleeding. Coagulation parameters such as low platelet count and certain comorbidities like chronic lung disease are potential targets to explore. The analysis of LOS or organ support-free days was limited by the small number of published studies. Ongoing and future RCTs are encouraged to explore these surrogate morbidity outcomes and their association with in-hospital mortality.

There are currently more than 40 ongoing RCTs that will further shed light on this topic. More recently, the American Society of Hematology published new guidance articles on thromboprophylaxis in COVID-19 patients with the following recommendations: (1) favor the use of prophylactic-intensity over intermediate-intensity anticoagulation in critically ill and noncritically ill COVID-19 patients, (2) favor the use of prophylactic-intensity over therapeutic-intensity anticoagulation in critically ill COVID-19 patients, and (3) favor the use of therapeutic-intensity anticoagulation over prophylactic-intensity anticoagulation in noncritically ill COVID-19 patients. 66 67 68 Hospital systems are advised to remain circumspect regarding the use of therapeutic anticoagulation in COVID-19 patients without confirmed or suspected VTE. An individualized assessment of patient's bleeding risk will ultimately impact the true clinical benefit of anticoagulation in each patient.
