# Supplementary material for: Does High-Dose Thromboprophylaxis Improve Outcomes in COVID-19 Patients? A Meta-analysis of Comparative Studies
Source: TH Open. 2022 Oct 19;6(4):e323–34. doi: 10.1055/a-1930-6492 (PMC9581586; doi:10.1055/a-1930-6492)
Supplement: Supplementary file 1 — Supplementary Material [file 10-1055-a-1930-6492-s220017.pdf]

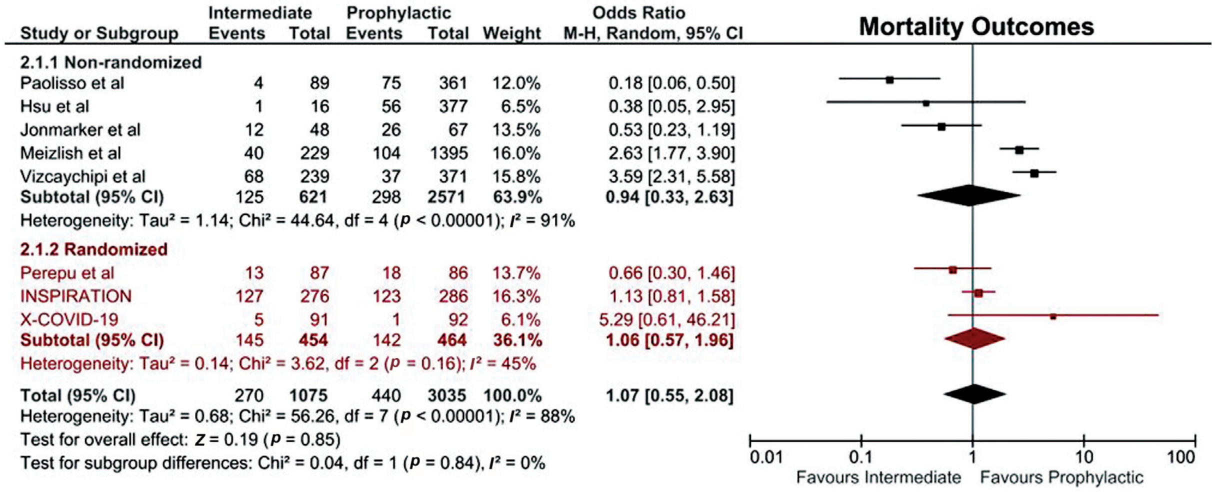

Supplementary Fig. S1 Mortality outcomes: intermediate versus prophylactic anticoagulation.

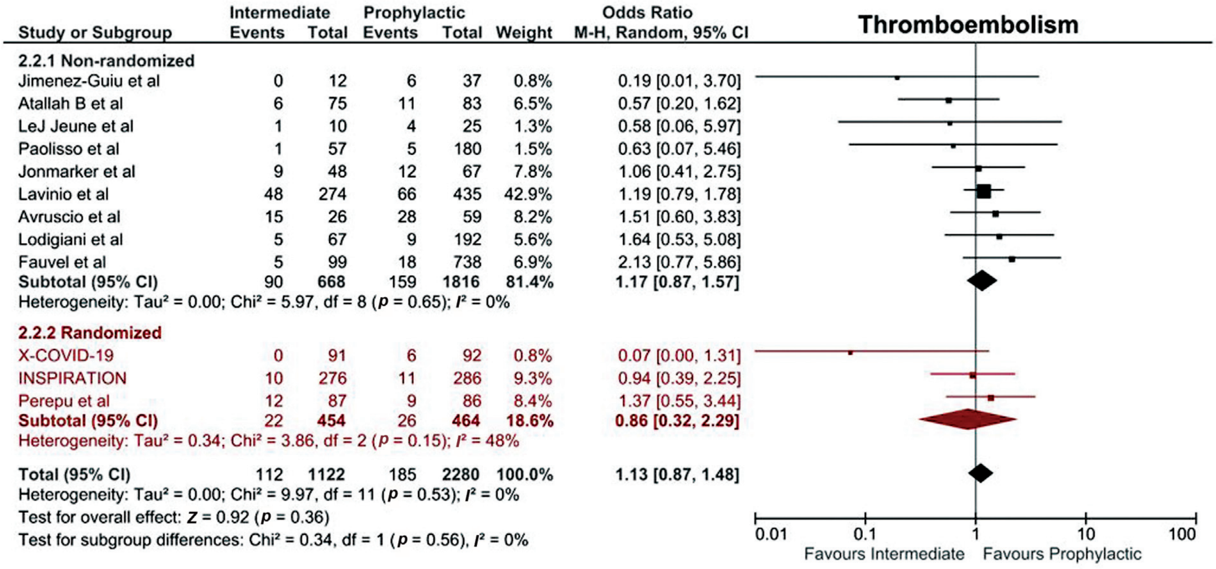

Supplementary Fig. S2 Thromboembolism risk: intermediate versus prophylactic anticoagulation.

 © 2022. The Author(s).

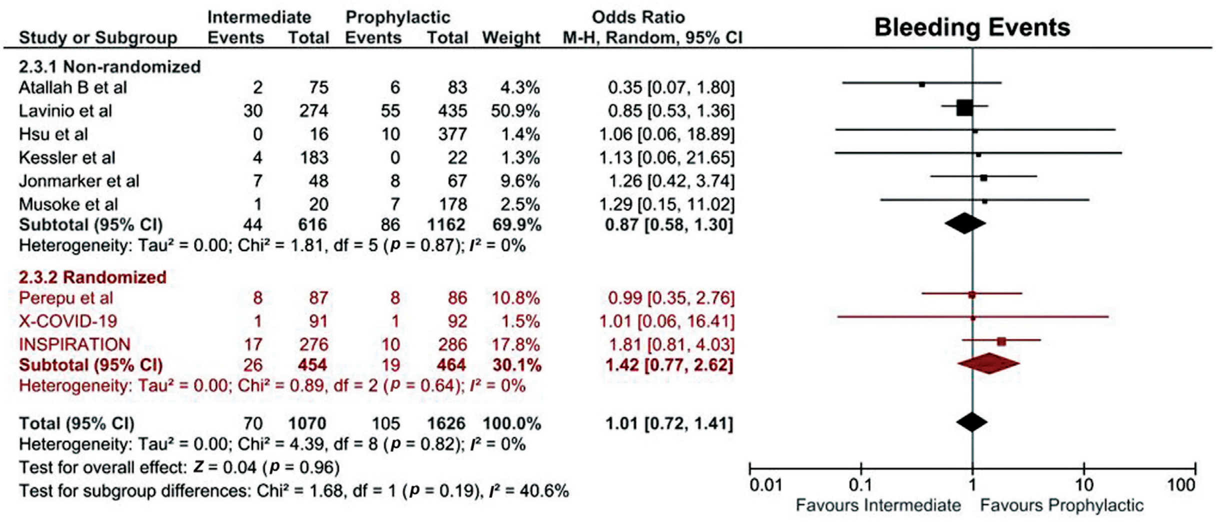

Supplementary Fig. S3 Bleeding rates: intermediate versus prophylactic anticoagulation.

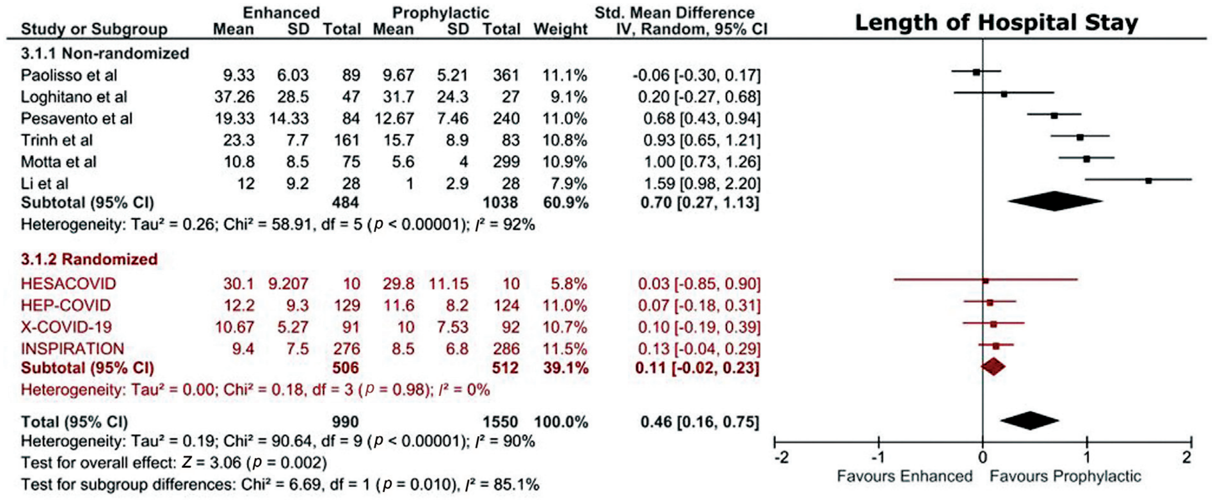

Supplementary Fig. S4 Length of hospital stay enhanced versus prophylactic.

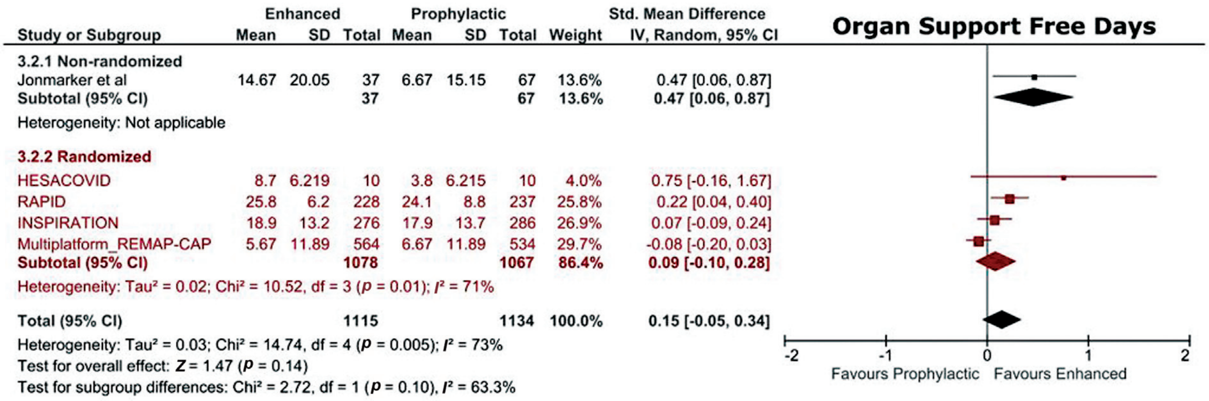

Supplementary Fig, S5 Organ support-free days: enhanced versus prophylactic.

Supplementary Table S1 Characteristics of included studies and thromboprophylaxis strategies

Study	Study design	Country, year	No. of patients (*n*)	ICU admits (%)	Prophylactic-dose group		Intermediate-dose group		Therapeutic-dose group	
					Type/dose	*n* (%)	Type/dose	*n* (%)	Type/dose	*n* (%)
ACTION (Lopes et al[43])	Pragmatic open label RCT	Brazil, 2021	614	6.3	Enoxaparin or UFH	304 (49.5)	NA	NA	Rivaroxaban (20 mg, or 15 mg), enoxaparin 1 mg/kg BID, unfractioted heparin	310 (50.6)
Al-Samkari et al[3]	Retrospective cohort	The United States, 2020	400	36	NA	354 (88.5)	NA	NA	NA	35 (8.75)
Al-Samkari et al[21]	Multicenter observational	The United States, 2021	,	100	Enoxaparin 40 mg q24 or UFH 5,000U q8	2,425 (86.3)	NA	NA	UFH, enoxaparin, bivaluridin, argatroban, others	384 (13.67)
Atalla et al[22]	Retrospective cohort	The United States, 2020	111	NA	Enoxaparin	53 (47.7)	NA	NA	DOAC and warfarin	17 (15.3)
Atallah et al[23]	Retrospective cohort	The United States, 2020	188	100	Enoxaparin 40 mg q24	83 (44.1)	Enoxaparin/UFH	75 (40)	Heparin drip	24 (12.8)
Avruscio et al[24]	Prospective cohort	Italy, 2020	85	48	Enoxaparin 40 mg q24 or fondaparinux 2.5 mg q24	59 (69.4)	Enoxaparin 60 mg or fondaparinux 5 mg q24	26 (30.6)	NA	NA
BEMICOP[59]	Multicenter, open label RCT	Spain, 2021	65	0	Bemiparin, 3,500 IU daily	33 (50.7)	NA	NA	Bemiparin, 115 IU/kg daily	49.2
Bolzetta et al[7]	Retrospective cohort	Italy, 2020	81	NA	Calciparin, fondaparinux, enoxaparin	57 (70.3)	NA	NA	Calciparin, fondaparinux, enoxaparin	24 (29.6)
Canoglu and Saylan[25]	Retrospective cohort	Turkey, 2020	154	48	LMWH 0.5 mg/kg q12	98 (63.6)	NA	NA	LMWH 1 mg/kg q12	56 (36.4)
Chistolini et al[26]	Case series	Italy, 2020	27	100	LMWH 100 IU/kg q24	14 (51.9)	NA	NA	LMWH 100 IU/kg BID	13 (48.1)
Cohen et al[8]	Retrospective cohort	The United States, 2020	9,407	20	Fondaparinux <7.5 mg q24, apixaban <10 mg q24, rivaroxaban <20 mg q24, enoxaparin < 80 mg q24	6,675 (71.0)	NA	NA	Fondaparinux >7.5 mg q24, apixaban >10 mg q24, rivaroxaban >20 mg q24, enoxaparin > 80 mg q24, dabigatran 150 mg q12, warfarin	1,753 (18.6)
Castelnuovo et al[27]	Retrospective single center	Italy, 2021	2,574	13	Heparin, 2,000 IU/day	983	NA	NA	Heparin, 6,000 IU/day	418
Fauvel et al[29]	Retrospective cohort	France, 2020	1,240	15	NA	738 (63.0)	NA	99 (7.9)	DOAC, VKA, heparin	136 (10.9)
Ferguson et al[30]	Retrospective cohort	The United States, 2020	141	100	Enoxaparin 40 mg q24 or 30 mg q12 or 0.5 mg/kg q12, Heparin 5,000U q12 or q8	95 (67.4)	NA	NA	Heparin drip or sq 1 mg/kg q12 or 1.5 mg/kg q24	46 (32.6)
Hanif et al[31]	Retrospective cohort	The United States, 2020	921	NA	NA	672 (73)	NA	NA	Unfractionated heparin, enoxaparin, DOACs	224 (24.3)
HEP-COVID[60]	Multicenter, double blind RCT	The United States, 2021	253	32.8	Enoxaparin, ≤40 mg daily	76 (30)	Enoxaparin 30-40 mg BID	48 (18.9)	Enoxaparin, 1 mg/kg SQ BID if CrCl > 30, or 0.5 mg/kg BID if CrCl was 15–29	129 (50.9)
HESA-COVID (Lemos et al)[39]	Phase II open label RCT	Brazil, 2020	20	100	UFH 5,000–7,500U q8 or enoxaparin 40 mg q24 (<120 kg) or 40 mg q12 (>120 kg)	10 (50)	NA	NA	Enoxaparin 1 mg/kg q12, 0.75 mg/kg q12 or 1 mg/kg q24 according to CrCl	10 (50)
Hsu et al[32]	Retrospective cohort	The United States, 2020	468	29		377 (80.5)		16 (3.4)		48 (10.28)

(*Continued*)

Supplementary Table S1 (*Continued*)

Study	Study design	Country, year	No. of patients (*n*)	ICU admits (%)	Prophylactic-dose group		Intermediate-dose group		Therapeutic-dose group	
					Type/dose	*n* (%)	Type/dose	*n* (%)	Type/dose	*n* (%)
					Enoxaparin 40 mg q24, UFH 5,000U q8 or apixaban 2.5 mg q12		Enoxaparin 40 mg q12 or UFH 7,500U q8		Enoxaparin 1 mg/kg q12, apixaban 5 mg q12, rivaroxaban 20 mg q24, or warfarin	
INSPIRATION (Sadeghipour et)[53]	Multicenter RCT, factorial design	Iran, 2021	562	100	Enoxaparin (or UFH if CKD), 40 mg qd	286 (50.8)	Enoxaparin (or UFH if CKD), 1 mg/kg qd	276 (49.1)	NA	NA
Jimenez-Guiu et al[33]	Prospective non-randomized cohort	Spain, 2020	57	0	Enoxaparin 40 mg q24	37 (64.9)	Enoxaparin 0.5 mg/kg q12	12 (21.1)	Enoxaparin 1.5 mg/kg q24	14
Jonmarker et al[4]	Retrospective cohort	Sweden, 2020	152	100	Tinzaparin 2,500–4,500IU or Dalteparin 2,500–5,000 IU daily	67 (44)	Tinzaparin >4,500 IU but <175 IU/kg or Dalteparin >5,000 IU but <200 IU/kg	48 (31.5)	Tinzaparin ≥175 IU/kg or dalteparin ≥200 IU/kg	37 (24.34)
Kessler et al[34]	Retrospective cohort	Switzerland, 2020	270	27	Enoxaparin 40 mg q24 or UFH 5,000 IU q12	22 (8.2)	Enoxaparin 40 mg q12 (<80 kg), or 60–80 mg q12 (≥80 kg) or UFH 5,000 IU TID	183 (67.8)	Enoxaparin (wt adjusted), UFH drip (anti-Xa 0.3–0.7 U/ml), standard dose DOAC or warfarin	65 (24)
Klok et al[35]	Retrospective cohort	Netherlands, 2020	184	100	Nandroparin 2,850 or 5,700/day or q12	167 (90.8)	NA	NA	NA	17 (9.2)
Koleilat et al[65]	Retrospective case-control	The United States, 2020	135	0	Enoxaparin/SQ heparin/apixaban	86 (63.7)	NA	NA	UFH/DOAC/bivalirudin	23
Lavinio et al[5]	Multicenter retrospective cohort	Europe	852	100	Enoxaparin/UFH	435	Enoxaparin/UFH, 40–80 mg twice daily	274	NA	NA
Le Jeune et al[37]	Retrospective cohort	France, 2020	42	7	NA	25 (59.5)	NA	10 (23.8)	NA	7 (16.7)
Lynn et al[36]	Retrospective cohort	The United States, 2020	402	27	NA	250 (62.1)	NA	NA	Heparin drip, Enoxaparin 1 mg/kg BID or 1.5 mg/kg daily, DOAC	152 (37.8)
Li et al	Retrospective cohort	The United States, 2020	56	43	UFH 5,000 U q8 or q12, enoxaparin 40 mg q24	28 (50)	NA	NA	UFH 8.4 ± 2.1U/kg/hr 15.1 ± 4 U/kg/hr	28 (50)
Llitjos et al[40]	Retrospective cohort	France, 2020	26	100	NA	8 (30.7)	NA		UFH, enoxaparin, LMWH	18 (69)
Lodigiani et al[41]	Retrospective cohort	Italy, 2020	388	16	Enoxaparin	192 (49.5)	NA	67 (17.2)	NA	76 (19.58)
Longhitano et al[42]	Prospective observational	Italy, 2020	74	24	Enoxaparin 80 U/kg q24, heparin 5,000U q8 or fondaparinux 2.5 mg q24	27 (36.4)	Enoxaparin <200 but >80 U/kg/day, UFH >15,000 but <25,000 U/day, fondaparinux 5 mg q24	24 (32.4%)	Enoxaparin 100 U/kg q12, heparin 12,500 U q12 or q8	23 (31)
Lonescu and Jaiyesimi	Retrospective cohort	The United States, 2020	3,480	19	UFH 5,000U q8 or q12, enoxaparin 30-40 mg q24, fondaparinux 2.5 mg daily	2,121 (60.9)	NA	NA	Apixaban, warfarin, rivaroxaban, dabigatran, UFH, fondaparinux, enoxaparin	998 (28.67)
Meizlish et al[45]	Retrospective cohort	Yale, 2020	2,785	34		1,395 (50.1)		229		531

 © 2022. The Author(s).

Supplementary Table S1 (*Continued*)

Study	Study design	Country, year	No. of patients (*n*)	ICU admits (%)	Prophylactic-dose group		Intermediate-dose group		Therapeutic-dose group	
					Type/dose	*n* (%)	Type/dose	*n* (%)	Type/dose	*n* (%)
					Enoxaparin 30–40 mg q24h, UFH 5,000-7,500u q8h with BMI ≥40 kg/m^2		Enoxaparin 0.4–0.7 mg/kg q12 h or UFH 7500 U with BMI < 40 kg/m^2		Enoxaparin ≥0.7 mg/kg q12 or ≥1.4 mg/kg q24h, IV UFH, or IV bivalirudin	
Middeldorp et al[46]	Retrospective cohort	Netherlands, 2020	198	38	Nadroparin 2,850 IU q24 for <100 kg or q12 or 5,700 IU for ≥100 kg	167 (84)	NA	NA	NA	19 (9.6)
Moll et al[47]	Retrospective cohort	The United States, 2020	210	49	UFH 5,000 IU q24 or Enoxaparin 40 mg sq q24	169 (80.5)	NA	NA	NA	21 (10)
Motta et al[48]	Retrospective cohort	The United States, 2020	374	17	Enoxaparin 30–40 mg sq q24 or heparin 5,000U q8	299 (79.9)	NA	NA	Enoxaparin 1 mg/kg q12 or 1.5 mg/kg q24 or heparin titrated according to aPTT 70-110 s	75 (20)
Multiplatform_ ATTACC[18]	Phase III, open-label RCT	Multi-national	2,244	0	LMWH or UFH	613 (71.7)	LMWH or UFH	227 (26.5)	LMWH or UFH	1,181 (52.6)
Multiplatform_ REMAP-CAP[18]	Phase III, open-label RCT	Multi-national	1,098	100	LMWH or UFH	199 (40.4)	LMWH or UFH	255 (51.7)	LMWH or UFH	564 (51.3)
Musoke et al[49]	Retrospective cohort	The United States, 2020	355	25	Heparin 5,000 q8–q12, enoxaparin 30–40 mg q24	178 (50.1)	Any dose in between	20 (5)	Enoxaparin 1 mg/kg q12, Heparin 80 U/kg bolus then 18 U/kg/hr or 12 U/kg/hr	102 (29)
Nadkarni et al[28]	Retrospective cohort	The United States, 2020	4,389	NA	Enoxaparin q24, or apixaban (2.5 mg q12 or 5 mg q24 in patients ≤75 years	1,959 (44.6)	NA	NA	Enoxaparin 1 mg/kg q12 or 1.5 mg/kg q24, apixaban 5 mg q12 or equivalent DOAC	900
Paolisso et al[50]	Retrospective cohort	Italy, 2020	450	15	Enoxaparin 40 mg–60 mg q24	361 (80.2)	Enoxaparin 40–60 mg q12	89 (19.7)	NA	NA
Paranjpe et al[51]	Retrospective cohort	The United States, 2020	2,773	14	NA	1,987 (71.6)	NA	NA	NA	786 (28)
Perepu et al[52]	Open label RCT	The United States, 2021	173	61.8	Enoxaparin, 40 mg sq daily if BMI < 30; 30 mg sq BID or 40 mg sq BID if BMI > 30	86 (49.7)	1 mg/kg sq daily if BMI <3 0; 0.5 mg/kg sq BID if BMI > 30	87 (50.3)	NA	NA
Pesavento et al[17]	Retrospective cohort	Italy, 2020	324	9	UFH up to 15,000 U/day, enoxaparin up to 4,000U/day and fondaparinux up to 2.5 mg/day	240 (74.1)	NA	NA	Unfractionated heparin, enoxaparin, fondaparinux	84 (26)
RAPID[61]	Multicenter RCT	Multiple	465	0	UFH or LMWH	237/465	NA	NA	UFH or LMWH	228/465
Pierce-Williams et al[64]	Retrospective cohort	The United States, 2020	64	30	Enoxaparin/heparin	37 (57.8)	NA	NA	Enoxaparin/heparin	10 (15.6)
Sadeghipour et al[53]	Multicenter RCT, factorial design	Iran, 2021	562	100	Enoxaparin (or ufh if ckd), 40 mg qd	286 (50.8)	Enoxaparin (or ufh if ckd), 1 mg/kg qd	276 (49.1)	NA	NA
Secco et al[54]	Retrospective cohort	Italy, 2020	115	100	Fondaparinux 2.5 mg q24, or enoxaparin 2000 to 6,000 IU/day	64 (55.6)	NA	NA	Enoxaparin > 8,000 IU daily, DOAC or warfarin of equivalent dose	48 (41.7)
Shah et al[55]	Retrospective cohort	The United Kingdom, 2020	187	100	Enoxaparin or UFH	151 (80.7)	NA	NA		31 (16.6)

(*Continued*)

Supplementary Table S1 (*Continued*)

Study	Study design	Country, year	No. of patients (*n*)	ICU admits (%)	Prophylactic-dose group		Intermediate-dose group		Therapeutic-dose group	
					Type/dose	*n* (%)	Type/dose	*n* (%)	Type/dose	*n* (%)
									Enoxaparin or UFH or DOAC or VKA	
Trinh et al[56]	Retrospective cohort	The United States, 2020	244	100	Enoxaparin, UFH 5,000 U, apixaban	83 (34)	NA	NA	Enoxaparin 1 mg/kg q12, heparin (PTT 70-100) or argatroban, bivalirudin, sq heparin, apixaban of equivalent dose	161 (65.98)
Vizcaychipi et al[9]	Prospective observational	The United Kingdom, 2020	939	14	Enoxaparin 40 mg q24	371 (39.5)	Enoxaparin 40 mg q12	239 (25.5)	Enoxaparin therapeutic dose	329 (35)
Voicu et al[57]	Exploratory study	France, 2020	93	100	Enoxaparin 40 mg q24 or UFH 15,000 IU/day if CrCl <15 mL/min	50 (53.7)	NA	NA	Enoxaparin 40 mg q12, 1 mg/kg q12 or UFH to reach plasma anti-Xa 0.3-0.6 IU/mL	43 (46.2)
Wei et al	Pragmatic cohort study	China 2021	749	38	LMWH 3,000–5,000 u/day	109/186	NA	NA	LMWH 100 u/kg q12hr	77/186
X-COVID-19[63]	Multicenter RCT	Italy, 2022	183	0	Enoxaparin, subcutaneous 40 mg o.d	92/183	Enoxaparin, subcutaneous 40 mg b.i.d		NA	NA

Abbreviations: BMI, Body mass index; DOAC, Direct oral anticoagulants; LMWH, Low molecular weight heparin; NA, Not available; RCT, Randomized controlled trial; sq, subcutaneous; UFH, Unfractionated heparin; VKA, Vitamin K antagonists; Wt, weight.

 © 2022. The Author(s).

Supplementary Table S2 Univariable analysis of study characteristics associated with risk of thromboembolism among nonrandomized studies

Covariate	Coefficient (β)	95%CI lower	95%CI upper	Two-sided *p*-value	R^2 analog	No. of studies
% ICU	−0.015	−0.027	−0.004	0.009	0.38	18
% Male	−0.049	−0.102	0.005	0.074	0.19	18
Age_median	0.004	−0.104	0.111	0.950	0	18
BMI_median	0.197	−0.349	0.743	0.479	0	11
% Obesity	−0.056	−0.134	0.022	0.159	0.29	5
FUdur	0.033	0.008	0.059	0.010	0.52	16
Dimer_median	−0.517	−1.357	0.323	0.228	0.09	9
Plt_median	−0.013	−0.037	0.010	0.271	0.1	3
Fibrinogen_median	−0.146	−0.618	0.326	0.543	0	11
% Cancer	0.084	−0.026	0.193	0.133	0.08	13
% HTN	0.011	−0.036	0.058	0.646	0	12
% CVS	0.025	−0.013	0.063	0.196	0	13
% Resp	0.017	−0.075	0.108	0.722	0	10
% CKD	0.025	−0.265	0.314	0.867	0	9
% DM	0.043	−0.016	0.102	0.149	0	13
% Aspirin	0.037	−0.093	0.167	0.574	0	6

Abbreviations: CI, confidence interval; CKD, Chronic kidney disease including dialysis patients; CVS, Cardiovascular disease; DM, Diabetes Mellitus; FUdur, follow up duration; HTN, hypertension; ICU, Intensive care unit; Plt, platelets; Resp, Respiratory disease (e.g. Asthma or COPD).

Supplementary Table S3 Univariable analysis of study characteristics associated with bleeding events among nonrandomized studies

Covariate	Coefficient (β)	95%CI lower	95%CI upper	Two-sided *p*-value	R^2 analog	No. of studies
% ICU	-0.001	-0.012	0.009	0.805	0	20
% Male	-0.030	-0.078	0.019	0.237	0	20
Age_median	0.009	-0.075	0.093	0.838	0	20
BMI_median	0.219	-0.156	0.593	0.253	0.07	14
% Obesity	0.005	-0.019	0.028	0.706	0	5
FUdur	-0.006	-0.042	0.031	0.754	0	15
Dimer_median	0.000	-0.590	0.589	1.000	0	9
Plt_median	-0.022	-0.051	0.006	0.124	0.04	9
Fibrinogen_median	-0.571	-1.503	0.362	0.231	0.19	11
% Cancer	0.082	-0.089	0.252	0.349	0	12
% HTN	0.026	-0.013	0.066	0.192	0.24	14
% CVS	0.007	-0.025	0.040	0.658	0	17
% Resp	0.059	0.000	0.118	0.051	0.51	15
% CKD	0.009	-0.087	0.105	0.857	0	12
% DM	0.040	-0.005	0.086	0.081	0.23	18
% Aspirin	0.038	-0.061	0.136	0.453	0	6

Abbreviations: CI, confidence interval; CKD, Chronic kidney disease including dialysis patients; CVS, Cardiovascular disease; DM, Diabetes Mellitus; FUdur, follow up duration; HTN, hypertension; ICU, Intensive care unit; Plt, platelets; Resp, Respiratory disease (e.g. Asthma or COPD).

 © 2022. The Author(s).

Supplementary Table S4 Quality assessment via New Castle–Ottawa Scale (NOS)

Study	Selection				Comparability	Outcome			Score	Quality
	Representativeness of the average adult in community	Cohort size	Information on clinical outcome	Outcome not present at start	Factors comparable between the groups	Adequate clinical assessment	Follow-up time	Adequacy of follow-up	MAX = 8	High > 5, medium > 3 but ≤ 5, low ≤ 3
	Population based: 1; multicenter: 0.5; single center: 0	>40 patients: 1; 39–20: 0.5; <20: 0	Information with clarity: 1; Information derived from percentage value: 0.5; unclear: 0	Not present: 1; present/na: 0	Yes: 1; no/na: 0	Yes: 1; no: 0	Yes: 1; not mentioned: 0	All patients followed up: 1; >50% followed up: 0.5; <50% followed-up or not mentioned: 0		
AlSamkari et al[3]	0.5	1	0	0	0	0	1	0.5	3	Low
Al Samkari et al[21]	0.5	1	1	1	0	1	1	1	6.5	High
Atalla t al[22]	0	1	0	1	0	1	0	1	4	Medium
Atallah B et al[23]	0	1	1	1	1	1	1	0	6	High
Avruscio et al[24]	0	1	1	1	0	1	0	1	5	Medium
Bolzetta et al[7]	0	1	0.5	1	1	1	1	1	6.5	High
Canoglu and Saylan[25]	0	1	1	1	1	1	0	1	6	High
Chistolini et al[26]	0	0.5	1	1	1	1	0	1	5.5	High
Cohen et al[8]	0	1	1	1	0	1	0	1	5	Medium
Castelnuovo[27]	0.5	1	0.5	0	0	1	1	0.5	4.5	Medium
Fauvel et al[29]	0.5	1	1	1	0	1	0	1	5.5	High
Ferguson et al[30]	0	1	0.5	1	1	1	1	0	5.5	High
Hanif et al[31]	0	1	1	1	1	1	0	0	5	Medium
Hsu et al[32]	0	1	1	1	1	1	1	1	7	High
Jimenez-Guiu et al[33]	0	1	0.5	1	0	1	1	1	5.5	High
Jonmarker et al[4]	0.5	1	1	1	1	1	1	1	7.5	High
Kessler et al[34]	0	1	1	1	1	1	0	1	6	High
Klok et al[35]	0.5	1	0.5	1	0	1	1	1	6	High
Koleilat et al[65]	0	1	1	0	0	0	1	0	3	Low
Lavinio et al[5]	0.5	1	1	1	0	1	0	0	4.5	Medium
Le Jeune et al[37]	0	1	1	1	1	1	1	1	7	High
Lynn et al[36]	0	1	1	1	1	1	0	1	6	High
Lemos et al[39]	0	0.5	1	1	1	1	1	1	6.5	High
Li et al	0	1	1	0	0	1	1	1	5	Medium
Llitjos et al[40]	0	0.5	1	1	1	1	1	1	6.5	High

 © 2022. The Author(s).

Supplementary Table S4 (*Continued*)

Study	Selection				Comparability	Outcome			Score	Quality
	Representativeness of the average adult in community	Cohort size	Information on clinical outcome	Outcome not present at start	Factors comparable between the groups	Adequate clinical assessment	Follow-up time	Adequacy of follow-up	MAX = 8	High > 5, medium > 3 but ≤ 5, low ≤ 3
	Population based: 1; multicenter: 0.5; single center: 0	>40 patients: 1; 39–20: 0.5; <20: 0	Information with clarity: 1; Information derived from percentage value: 0.5; unclear: 0	Not present: 1; present/na: 0	Yes: 1; no/na: 0	Yes: 1; no: 0	Yes: 1; not mentioned: 0	All patients followed up: 1; >50% followed up: 0.5; <50% followed-up or not mentioned: 0		
Lodigiani et al[41]	0	1	1	1	0	1	1	1	6	High
Lonescu and Jaiyesimi	0.5	1	1	0	0	1	1	1	5.5	High
Longhitano et al[42]	0	1	1	1	1	1	1	1	7	High
Meizlish et al[45]	0.5	1	1	1	1	1	0	1	6.5	High
Middeldorp et al[46]	0	1	1	0	0	1	1	0	4	Medium
Moll et al[47]	0	1	1	1	1	1	1	1	7	High
Motta et al[48]	0	1	1	1	0	1	1	1	6	High
Musoke et al[49]	0	1	1	1	0	1	0	0	4	Medium
Nadkarni et al[28]	0.5	1	1	1	1	1	0	1	6.5	High
Paolisso et al[50]	0	1	1	1	1	1	1	1	7	High
Paranjpe et al[51]	0	1	0.5	0	0	1	1	1	4.5	Medium
Pesavento et al[17]	0.5	1	1	1	0	1	1	1	6.5	High
Pierce Williams et al[64]	0.5	1	0	1	0	0	0	0	2.5	Low
Secco et al[54]	0	1	1	1	0	1	1	1	6	High
Shah et al[55]	0.5	1	1	0	0	1	1	1	5.5	High
Trinh et al[56]	0	1	0.5	1	1	1	1	1	6.5	High
Vizcaychipi et al[9]	0.5	1	1	0	0	1	0	1	4.5	Medium
Voicu et al[57]	0	1	1	0	1	1	0	1	5	Medium
Wei et al	0	1	0	0	1	1	1	0	4	Medium

 © 2022. The Author(s).

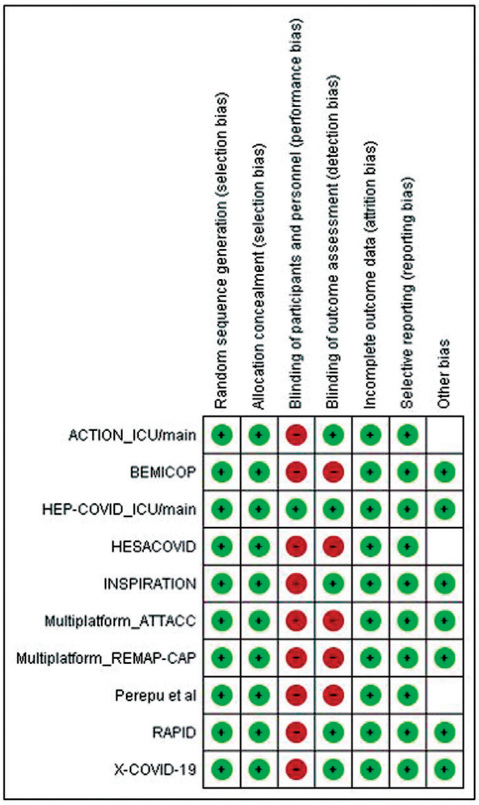

Supplementary Fig. S6 Risk of bias summary figure among RCTs. RCT, randomized controlled trial.

 © 2022. The Author(s).
